# Neutrophil Percentage–to-Albumin Ratio as a Novel Prognostic Biomarker in Adult Diffuse Gliomas: Retrospective Study Integrating 3 Machine Learning Models and Cox Regression

**DOI:** 10.2196/79945

**Published:** 2026-01-13

**Authors:** Congcong Zhu, Jiyang An, Lili Zhou

**Affiliations:** 1 Department of Oncology The First Affiliated Hospital of Zhengzhou University Zhengzhou, Henan China; 2 Department of Oncology The First Affiliated Hospital, and College of Clinical Medicine of Henan University of Science and Technology Luoyang, Henan China; 3 Department of Neurosurgery The First Affiliated Hospital of Zhengzhou University Zhengzhou, Henan China

**Keywords:** adult-type diffuse glioma, machine learning, neutrophil percentage-to-albumin ratio, NPAR, prediction model, prognosis

## Abstract

**Background:**

Adult-type diffuse glioma (ADG) is the most common primary malignant tumor of the central nervous system. Its highly invasive nature, marked heterogeneity, and resistance to therapy contribute to a high risk of recurrence and poor prognosis. At present, the lack of reliable prognostic tools poses a significant barrier to the development of individualized treatment strategies.

**Objective:**

This study aimed to develop an effective prognostic model for ADG by integrating multiple machine learning algorithms, in order to enhance the precision of individualized clinical decision-making.

**Methods:**

In this retrospective study, 160 newly diagnosed patients with ADG who underwent surgical resection and histopathological confirmation at our institution between June 2019 and September 2021 were included. A total of 32 variables, including clinical characteristics, molecular biomarkers, and preoperative hematological indicators, were collected. Overall survival (OS) and progression-free survival (PFS) were defined as the study endpoints. Feature selection was performed using least absolute shrinkage and selection operator regression, extreme gradient boosting, and random forest algorithms. Kaplan-Meier survival curves and log-rank tests were used for survival analysis. Multivariate Cox proportional hazards models were constructed to identify independent prognostic factors, and nomograms were developed accordingly. The model’s discriminative ability, calibration, and clinical utility were evaluated using the concordance index, area under the receiver operating characteristic curve (area under the curve), calibration plots, and Kaplan-Meier analysis.

**Results:**

Age, neutrophil percentage–to-albumin ratio (NPAR), and platelet-to-mean platelet volume ratio were identified as independent prognostic factors for OS, while age and NPAR were independent predictors for PFS (all *P*<.001). The prognostic models based on these variables demonstrated good predictive performance, with concordance index values of 0.731 and 0.763 for the training and validation cohorts in the OS model, respectively. The PFS model also showed robust performance. Area under the curve values and calibration curves further supported the models’ accuracy and stability. Risk stratification analysis revealed clear survival differences between risk groups (all *P*<.05), indicating strong clinical applicability.

**Conclusions:**

This study is the first to identify preoperative NPAR as a significant prognostic biomarker for ADG using machine learning approaches. The prognostic model incorporating NPAR, platelet-to-mean platelet volume ratio, and age demonstrated favorable predictive performance, offering a novel perspective for accurate risk stratification and personalized treatment in patients with ADG.

## Introduction

Glioma is the most common primary malignant tumor of the central nervous system, with an annual incidence of approximately 4.5 per 100,000 individuals [1]. Among its subtypes, adult-type diffuse glioma (ADG) constitutes the predominant pathological category, characterized by high invasiveness, frequent postoperative recurrence, and poor overall survival (OS) [2]. Epidemiological data indicate that ADG incidence exhibits age and sex specificity, with the highest prevalence observed in individuals aged 45-70 years (peak incidence at age 65-75 years) and a male predominance (male-to-female ratio: 1.3-1.6:1) [3].

Despite advances in multimodal treatment—including maximal surgical resection combined with radiochemotherapy—ADG survival outcomes have shown limited improvement over the past decade [4,5]. Novel therapies such as immune checkpoint inhibitors (eg, anti–PD-1 monoclonal antibodies) and targeted agents (eg, epidermal growth factor receptor inhibitors) offer promise, but their clinical benefits are restricted to specific molecular subgroups and are frequently accompanied by immune-related adverse events, such as pneumonitis (reported in 15%-20% of cases) and acquired resistance [6]. As such, there remains an urgent need for precise prognostic tools to identify treatment-sensitive subpopulations and guide individualized therapeutic decision-making.

Currently, prognostic evaluation of ADG largely depends on traditional clinicopathological parameters, including age, isocitrate dehydrogenase 1 (IDH1) mutation status, and O6-methylguanine-DNA methyltransferase (MGMT) promoter methylation [7,8]. However, these markers alone exhibit limited predictive accuracy for treatment response or survival. Emerging evidence suggests that inflammation within the tumor microenvironment (TME) plays a critical role in glioma progression by promoting angiogenesis and suppressing antitumor immune activity [9]. Peripheral blood–derived inflammatory biomarkers, such as the neutrophil-to-lymphocyte ratio (NLR), lymphocyte-to-monocyte ratio (LMR), and platelet-to-lymphocyte ratio (PLR), have demonstrated prognostic relevance in several malignancies, including breast and colorectal cancer [10,11]. However, their utility in ADG remains underinvestigated.

Recently, the neutrophil percentage-to-albumin ratio (NPAR)—a composite marker integrating systemic inflammation and nutritional status—has been proposed as a novel prognostic indicator in solid tumors [12]. Nevertheless, its prognostic value in ADG, particularly in the context of long-term survival and treatment response, remains unclear.

Moreover, most existing prognostic models in gliomas are based on conventional Cox regression analysis, which is limited in handling high-dimensional data and complex nonlinear interactions [13,14]. In contrast, machine learning algorithms such as least absolute shrinkage and selection operator (LASSO) regression, extreme gradient boosting (XGBoost), and random forest (RF) have shown superior performance in variable selection and pattern recognition [15-18]. However, no studies to date have integrated these 3 algorithms to enhance the robustness of prognostic modeling in ADG.

Therefore, this study proposes an innovative machine learning–based approach that integrates LASSO regression (for feature selection), XGBoost (a gradient-boosted decision tree method), and RF (an ensemble learning model) to develop a multidimensional prognostic tool based on clinical, molecular, and inflammatory parameters. The objective is to improve predictive accuracy and provide refined support for clinical decision-making in patients with ADG.

## Methods

### Ethical Considerations

This retrospective study was conducted in accordance with the Declaration of Helsinki and was approved by the Ethics Committee of The First Affiliated Hospital of Zhengzhou University (Approval No 2023-KY-0223-002). Given the retrospective nature of this study, the requirement for informed consent was waived by the ethics committee. All patient data were anonymized and deidentified prior to analysis. Personal identifiers were removed, and each patient was assigned a unique study code to ensure confidentiality throughout the research process. No financial compensation was provided to the participants.

### Study Population

This retrospective study enrolled 160 treatment-naïve patients with ADG who underwent maximal safe surgical resection with histopathological confirmation at our institution between June 2019 and September 2021. The postoperative treatment regimen followed the standard protocol, consisting of concurrent chemoradiotherapy followed by adjuvant temozolomide chemotherapy. In cases of disease progression, targeted therapies such as bevacizumab or BRAF (B-Raf proto-oncogene, serine/threonine kinase) inhibitors (eg, for tumors harboring the BRAF V600E mutation) were administered based on clinical indications [19].

Inclusion criteria were as follows: (1) age ≥18 years, diagnosis of ADG in accordance with the 5th edition of the *WHO Classification of Tumors of the Central Nervous System* (World Health Organization [WHO] grade 3-4); (2) completion of preoperative magnetic resonance imaging or computed tomography evaluation; (3) no prior antitumor therapy; (4) receipt of standard treatment protocol; (5) availability of complete clinical data, including general characteristics, preoperative blood tests, cardiac, hepatic, and renal function assessments, as well as postoperative immunohistochemistry and molecular profiling; and (6) complete follow-up records.

Exclusion criteria were (1) postoperative mortality within 30 days due to surgical complications or nonneoplastic causes; (2) presence of severe systemic disease or active infection potentially affecting study outcomes; (3) diagnosis of other concurrent malignancies; (4) significant cardiac, hepatic, or renal dysfunction; and (5) history of prior cranial surgery.

Postoperative follow-up included clinical and radiological evaluations every 3 months during the first year and every 6 months thereafter, until October 1, 2024. Progression-free survival (PFS) was defined as the time from diagnosis to radiographic or clinical disease progression or death from any cause. OS was defined as the time from diagnosis to death or the last follow-up.

### Study Variables

Clinical data were collected from patients with ADG who met the inclusion and exclusion criteria. The variables analyzed in this study included: clinical characteristics, molecular biomarkers, genetic alteration markers, preoperative peripheral hematological indicators, and inflammation-related ratios.

#### Clinical Characteristics

Age, gender, preoperative tumor location, extent of tumor resection, maximum tumor diameter, and midline shift.

#### Molecular Biomarkers

Immunohistochemical markers included P53 protein (P53), oligodendrocyte transcription factor 2 (Olig-2), S100 calcium-binding protein (S100), epithelial membrane antigen (EMA), synuclein (SYN), alpha-thalassemia/mental retardation syndrome X-linked (ATRX), cluster of differentiation 34 (CD34), neuronal nuclei (NeuN), glial fibrillary acidic protein (GFAP), and proliferation cell nuclear antigen-67 (Ki-67).

#### Genetic Alteration Markers

IDH1 immunophenotype, telomerase reverse transcriptase (TERT) promoter region mutation, BRAF V600E point mutation, epidermal growth factor receptor (EGFR) amplification, MGMT promoter methylation status, 1p loss of heterozygosity (1pLOH), and 19q loss of heterozygosity (19qLOH).

#### Preoperative Peripheral Hematological Indicators

White blood cell (WBC), platelet (PLT), neutrophil (Neut), monocyte (Mono), lymphocyte (Lym), prothrombin time (PT), activated partial thromboplastin time (APTT), lactate dehydrogenase (LDH), and serum albumin (ALB). All preoperative peripheral hematological indicators, including NPAR, were measured from blood samples collected within 1 week prior to surgery, before the administration of any corticosteroids or other interventions that could significantly alter these values.

#### Inflammation-Related Ratios

According to international standards for inflammatory biomarkers, the following parameters were calculated and defined: PLR = PLT/Neut, NLR = Neut/Lym, LMR = Lym/Mono, monocyte-to-lymphocyte ratio (MLR) = Mono/Lym, prognostic nutritional index (PNI) = ALB + 5 × Lym, systemic inflammation response index (SIRI) = (Neut × Mono)/Lym, systemic immune-inflammation index (SII) = (PLT × Neut)/Lym, aggregate index of systemic inflammation (AISI) = (Neut × PLT × Mono)/Lym, NPAR = (Neut/WBC%)/ALB, neutrophil-lymphocyte-platelet ratio (NLPR) = Neut/(Lym × PLT).

### Study Design and Statistical Analysis

**Figure 1:**
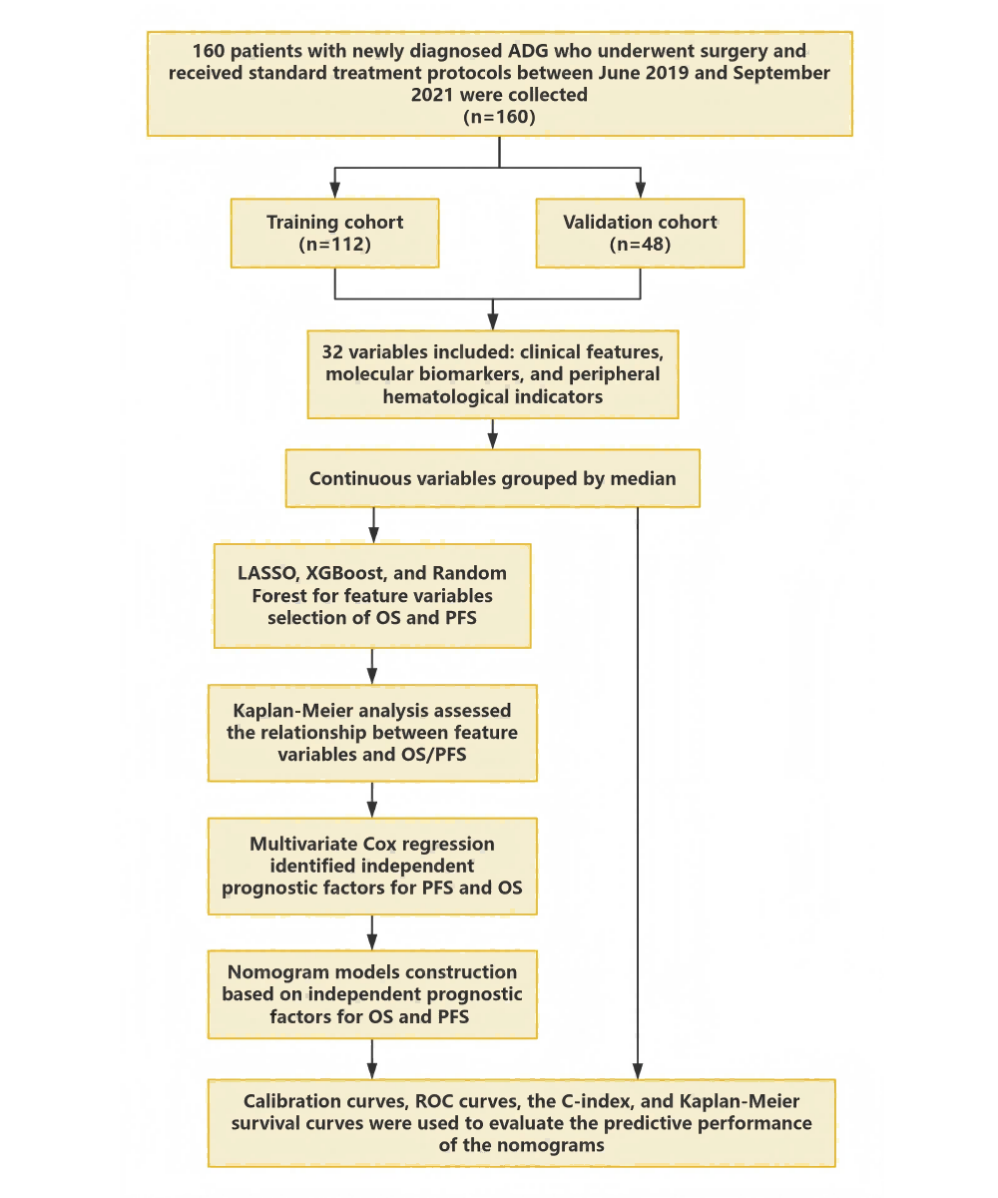
Flowchart of the study design.

The study design is illustrated in Figure 1.

A random seed was set to divide the 160 cases into a training cohort (n=112) and a validation cohort (n=48) at a 7:3 ratio. The primary endpoint was OS, and the secondary endpoint was PFS. A total of 32 variables were included, encompassing clinical characteristics (age, gender, tumor location, etc), molecular biomarkers (Ki-67, IDH1, R132H, etc), and inflammatory indicators (NPAR, NLR, etc).

Continuous variables were dichotomized using the median as the cutoff. This approach was chosen to simplify the clinical interpretation of the model and to facilitate risk stratification, while also mitigating the impact of potential outliers and meeting the model’s assumption of proportional hazards for categorical predictors. Categorical variables were expressed as frequencies (percentages), and group comparisons were conducted using the chi-square test (expected frequency ≥5) or Fisher exact test (expected frequency <5). The Shapiro-Wilk test was used to assess the normality of continuous variables. For normally distributed data, comparisons between groups were performed using the 2-tailed independent-samples *t* test (2 groups) or 1-way ANOVA (3 or more groups), with data presented as mean (SD). For nonnormally distributed data, comparisons were performed using the Mann-Whitney *U* test (2 groups) or the Kruskal-Wallis H test (3 or more groups), with post hoc pairwise comparisons adjusted using the Bonferroni method. These data were reported as median (IQR). A 2-tailed *P* value <.05 was considered statistically significant for all analyses.

A total of 3 machine learning algorithms were applied: LASSO regression (using the *glmnet* package with 10-fold cross-validation to optimize λ), XGBoost (using the *xgboost* package with max depth=6 and learning rate=0.01), and RF (using the *randomForest* package with *ntree*=1000). Venn diagrams were used to identify overlapping important variables selected by all 3 algorithms. Survival differences between groups were visualized using Kaplan-Meier (KM) curves and compared using the log-rank test.

A Cox proportional hazards regression model was constructed to estimate hazard ratios (HRs) and 95% CIs. Based on multivariate Cox results, a nomogram was developed using the *rms* package. Time-dependent receiver operating characteristic (ROC) curves were generated, and the area under the curve (AUC) at 1, 3, and 5 years was calculated. The concordance index (C-index) was used to evaluate the model’s discrimination ability. Calibration was assessed using calibration curves based on 1000 bootstrap resamples. KM survival analysis based on the median nomogram score was performed to evaluate clinical utility. The validation cohort was used to assess the model’s predictive performance.

All statistical analyses were conducted using R software (version 4.4.1; R Foundation for Statistical Computing). A 2-sided *P* value <.05 was considered statistically significant.

## Results

### Survival Outcomes

**Figure 2:**
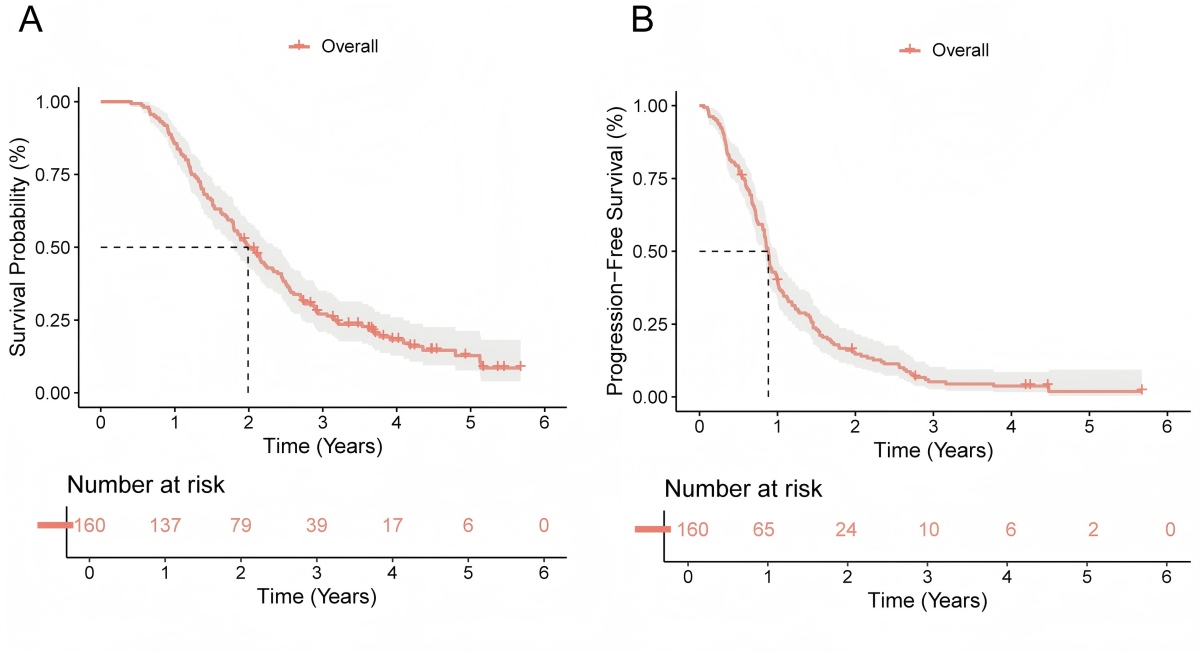
Postoperative survival curves of 160 patients with adult-type diffuse glioma: (A) overall survival (OS) and (B) progression-free survival (PFS).

Among the 160 patients included in this study, a total of 132 (82.5%) experienced death events, and 152 (95%) experienced disease progression. The median OS was 23.0 months (IQR 15.0-38.3 months), while the median PFS was 10.5 months (IQR 6.8-17.5 months). The 1-, 3-, and 5-year OS rates were 85.6%, 27.1%, and 12.8%, respectively. The corresponding 1-, 3-, and 5-year PFS rates were 40.0%, 6.5%, and 3.4%, respectively. KM survival curves are presented in Figure 2.

### Baseline Characteristics

A total of 160 patients were included in this study, with a male-to-female ratio of approximately 1.4:1. The mean age was 51.3 (SD 13.6) years, and the median age was 53 (IQR 44-61) years. The maximum tumor diameter ranged from 1.0 to 9.5 cm, with a mean of 4.7 (SD 1.7) cm and a median of 5.0 cm (IQR 3.4-5.9 cm). Tumors were predominantly unilateral, with 76 cases (47.5%) on the left, 80 (50%) on the right, and 2 (2.5%) involving both sides. Midline shift was observed in 59.4% (95/160) of patients. All cases were classified as WHO grade 3-4. No significant differences in baseline characteristics were found between the training and validation cohorts (all *P*>.05). Detailed information is provided in Table 1.

**Table 1:** Baseline characteristics of 160 patients with adult-type diffuse glioma (ADG) stratified by group.

Variable		Training group, n (%)	Validation group, n (%)	*P* value	
**Age (years)**					.58
	<53	56 (50)	21 (44)		
	≥53	56 (50)	27 (56)		
**Sex**					.06
	Male	71 (63.4)	22 (46)		
	Female	41 (36.6)	26 (54)		
**Maximum tumor diameter (cm)**					.89^a^
	<5.0	55 (49.1)	23 (47)		
	≥5.0	57 (50.9)	25 (52)		
**Location**					.74^b^
	Left	54 (48.2)	22 (46)		
	Right	56 (50.0)	24 (50)		
	Double	2 (1.8)	2 (4)		
**Midline shift**					.16
	No	41 (36.6)	24 (50)		
	Yes	71 (63.4)	24 (50)		
**Neutrophil percentage–to-albumin ratio**					.92^b^
	<1.578	55 (49.1)	24 (50)		
	≥1.578	57 (50.9)	24 (50)		
**Aggregate index of systemic inflammation**					.60
	<272.854	58 (51.8)	22 (46)		
	≥272.854	54 (48.2)	26 (54)		
**Immune-inflammation index**					.86
	<146.649	55 (49.1)	25 (52)		
	≥146.649	57 (50.9)	23 (48)		
**Lymphocyte-to-monocyte ratio**					.39
	<3.750	59 (52.7)	21 (44)		
	≥3.750	53 (47.3)	27 (56)		
**Neutrophil-to-lymphocyte ratio**					.12
	<2.677	51 (45.5)	29 (60)		
	≥2.677	61 (54.5)	19 (40)		
**Platelet-to-lymphocyte ratio**					.23
	<138.251	52 (46.4)	28 (58)		
	≥138.251	60 (53.6)	20 (42)		
**Monocyte-to-lymphocyte ratio**					.39
	<0.267	53 (47.3)	27 (56)		
	≥0.267	59 (52.7)	21 (44)		
**Prognostic nutritional index**					.47
	<50.55	52 (46.4)	26 (54)		
	≥50.55	60 (53.6)	22 (46)		
**Systemic inflammation response index**					.86
	<1.166	55 (49.1)	25 (52)		
	≥1.166	57 (50.9)	23 (48)		
**L** **actate dehydrogenase** **(IU/L)**					.54
	<174	53 (47.3)	26 (54)		
	≥174	59 (52.7)	22 (46)		
**Albumin** **(g/L)**					.12
	<42.35	51 (45.5)	29 (60)		
	≥42.35	61 (54.5)	19 (40)		
**Prothrombin time/Activated partial thromboplastin time**					.39
	<0.372	53 (47.3)	27 (56)		
	≥0.372	59 (52.7)	21 (44)		
**Platelet-to-mean platelet volume ratio**					.12
	<25.322	61 (54.5)	19 (40)		
	≥25.322	51 (45.5)	29 (60)		
**Proliferation cell nuclear antigen-67**					.16
	<30	28 (25)	18 (37)		
	≥30	84 (75)	30 (63)		
**Glial fibrillary acidic protein**					>.99^b^
	Positive	108 (96.4)	47 (98)		
	Negative	4 (3.6)	1 (2)		
**S100 calcium-binding protein**					.74^b^
	Positive	105 (93.8)	44 (92)		
	Negative	7 (6.2)	4 (8)		
**Oligodendrocyte transcription factor 2**					>.99^b^
	Positive	107 (95.5)	46 (96)		
	Negative	5 (4.5)	2 (4)		
**P53 protein**					.09
	Positive	103 (92)	39 (81)		
	Negative	9 (8)	9 (19)		
**Alpha-thalassemia/mental retardation syndrome X-linked**					.27
	Positive	96 (85.7)	37 (77)		
	Negative	16 (14.3)	11 (23)		
**Cluster of differentiation 34**					.66
	Positive	34 (30.4)	17 (35)		
	Negative	78 (69.6)	31 (65)		
**Epithelial membrane antigen**					.56^b^
	Positive	12 (10.7)	3 (6)		
	Negative	100 (89.2)	45 (94)		
**Synuclein**					.40
	Positive	22 (19.6)	13 (27)		
	Negative	90 (80.4)	35 (73)		
**Neuronal nuclei**					>.99^b^
	Positive	2 (1.9)	1 (2)		
	Negative	110 (58.1)	47 (98)		
**O6-methylguanine-DNA methyltransferase promoter methylation**					>.99^b^
	Methylated	6 (5.4)	2 (4)		
	Unmethylated	106 (94.6)	46 (96)		
**Isocitrate dehydrogenase 1 R132**					.78^b^
	Positive	12 (10.7)	4 (8)		
	Negative	100 (89.2)	44 (92)		
**1p loss of heterozygosity**					.07^b^
	Positive	2 (1.9)	4 (8)		
	Negative	110 (58.1)	44 (92)		
**19q loss of heterozygosity**					.74^b^
	Positive	7 (6.2)	4 (8)		
	Negative	105 (93.8)	44 (92)		

^a^Indicates without continuity correction.

^b^Indicates Fisher exact test.

### Differences in PFS and OS Between Groups

Since the measurement data did not meet the assumption of normality according to the Shapiro-Wilk test, the nonparametric Wilcoxon rank-sum test was applied to evaluate the impact of different clinical characteristics on PFS and OS. Data are presented as median (IQR).

Using PFS as the outcome variable, significant differences (all *P*<.05) were observed in the following 10 variables: age (*P*<.001), tumor location (*P*=.03), NPAR (*P*=.002), NLR (*P*=.002), PNI (*P*=.01), SYN (*P*=.003), MGMT promoter methylation (*P*=.004), IDH1 R132 (*P*<.001), 1pLOH (*P*=.04), and 19qLOH (*P*<.001).

Using OS as the outcome variable, a total of 12 variables showed significant associations (all *P*<.05): age (*P*<.001), NPAR (*P*<.001), LMR (*P*=.03), NLR (*P*=.02), MLR (*P*=.03), PNI (*P*=.01), LDH (*P*=.04), SYN (*P*=.009), MGMT (*P*=.04), IDH1 R132 (*P*<.001), 1pLOH (*P*=.003), and 19qLOH (*P*<.001). Detailed results are presented in Table 2.

**Table 2:** Comparison of progression-free survival (PFS) and overall survival (OS) among patients with glioblastoma between groups, median (IQR). The z and H values denote the test statistics for the Wilcoxon rank-sum test used in 2-group comparisons and the Kruskal-Wallis test used in multigroup comparisons, respectively.

Variable		Frequency, n	PFS (year), median (IQR)	z or H		*P* value		OS (year), median (IQR)		z or H		*P* value	
**Age (years)**					4.42^a^		<.001				6.67^a^		<.001
	<53	77	1.02 (0.74-2.37)					2.76 (1.96-3.67)					
	≥53	83	0.73 (0.36-1.07)					1.36 (1.03-2.07)					
**Sex**					0.49^a^		.63				0.16^a^		.88
	Male	93	0.90 (0.63-1.43)					2.03 (1.22-3.06)					
	Female	67	0.84 (0.52-1.61)					1.95 (1.29-2.90)					
**Maximum tumor diameter (cm)**					–1.14^a^		.25				–1.04^a^		.30
	<5.0	78	0.85 (0.59-1.45)					1.87 (1.21-2.91)					
	≥5.0	82	0.92 (0.54-1.49)					2.13 (1.33-3.02)					
**Location**					7.050^b^		.03				3.26 ^b^		.20
	Left	76	0.90 (0.65-1.54)					2.05 (1.35-2.89)					
	Right	80	0.86 (0.59-1.46)					1.98 (1.21-3.21)					
	Double	4	0.33 (0.27-0.41)					1.13 (0.82-1.62)					
**Midline-shift**					–0.54^a^		.59				–0.86^a^		.39
	No	65	0.85 (0.55-1.52)					1.80 (1.21-2.91)					
	Yes	95	0.90 (0.58-1.45)					2.09 (1.33-3.00)					
**Neutrophil percentage–to-albumin ratio**					2.86^a^		.004				3.73^a^		<.001
	<1.578	79	0.99 (0.67-1.74)					2.47 (1.53-3.58)					
	≥1.578	81	0.83 (0.36-1.18)					1.72 (1.03-2.44)					
**Aggregate index of systemic inflammation**					1.35^a^		.18				1.28^a^		.20
	<272.854	80	0.89 (0.67-1.47)					2.13 (1.32-3.38)					
	≥272.854	80	0.88 (0.39-1.46)					1.91 (1.26-2.71)					
**Immune-inflammation index**					1.69^a^		.09				0.68^a^		.498
	<146.649	80	0.89 (0.66-1.72)					2.07 (1.22-2.99)					
	≥146.649	80	0.83 (0.50-1.24)					1.95 (1.30-2.90)					
**Lymphocyte-to-monocyte ratio**					–0.59^a^		.56				–2.13^a^		.03
	<3.750	80	0.85 (0.53-1.43)					1.85 (1.22-2.52)					
	≥3.750	80	0.89 (0.59-1.54)					2.45 (1.33-3.49)					
**Neutrophil-to-lymphocyte ratio**					2.83^a^		.005				2.41^a^		.02
	<2.677	80	0.94 (0.67-1.81)					2.33 (1.35-3.56)					
	≥2.677	80	0.83 (0.35-1.24)					1.85 (1.15-2.52)					
**Platelet-to-lymphocyte ratio**					0.85^a^		.40				0.47^a^		.64
	<138.251	81	0.88 (0.61-1.54)					2.07 (1.22-3.13)					
	≥138.251	79	0.88 (0.48-1.44)					1.94 (1.30-2.92)					
**Monocyte-to-lymphocyte ratio**					0.59^a^		.56				2.13^a^		.03
	<0.267	80	0.89 (0.59-1.54)					2.45 (1.33-3.49)					
	≥0.267	80	0.85 (0.53-1.43)					1.85 (1.22-2.52)					
**Prognostic nutritional index**					–2.26^a^		.02				–3.27^a^		.001
	<50.55	78	0.83 (0.37-1.24)					1.79 (1.04-2.49)					
	≥50.55	82	0.98 (0.64-1.61)					2.35 (1.51-3.21)					
**Systemic inflammation response index**					1.63^a^		.10				1.82^a^		.07
	<1.166	80	0.90 (0.67-1.53)					2.39 (1.33-3.49)					
	≥1.166	80	0.84 (0.38-1.46)					1.87 (1.22-2.56)					
**Lactate dehydrogenase**					1.04^a^		.30				2.11^a^		.04
	<174	79	0.90 (0.51-1.79)					2.21 (1.34-3.21)					
	≥174	81	0.85 (0.60-1.19)					1.80 (1.22-2.61)					
**Albumin**					–0.62^a^		.54				–1.61^a^		.11
	<42.35	80	0.85 (0.64-1.27)					1.80 (1.21-2.63)					
	≥42.35	80	0.92 (0.50-1.70)					2.15 (1.38-3.07)					
**Prothrombin time/activated partial thromboplastin time**					–0.12^a^		.91				0.52^a^		.60
	<0.372	80	0.87 (0.51-1.47)					1.98 (1.21-3.56)					
	≥0.372	80	0.88 (0.61-1.41)					2.00 (1.38-2.75)					
**Platelet-to-mean platelet volume ratio**					1.03^a^		.30				0.10^a^		.92
	<25.322	80	0.88 (0.66-1.68)					1.96 (1.29-2.92)					
	≥25.322	80	0.87 (0.50-1.31)					2.01 (1.26-2.97)					
**Proliferation cell nuclear antigen-67**					1.08^a^		.28				1.33^a^		.19
	<30	46	0.92 (0.62-1.72)					2.36 (1.37-3.79)					
	≥30	114	0.87 (0.52-1.43)					1.92 (1.24-2.74)					
**Glial fibrillary acidic protein**					0.21^a^		.84				1.09^a^		.28
	Positive	155	0.88 (0.58-1.46)					1.96 (1.25-2.92)					
	Negative	5	1.28 (0.21-2.76)					2.55 (2.22-4.35)					
**S100 calcium-binding protein**					–0.57^a^		.57				0.37^a^		.71
	Positive	149	0.88 (0.59-1.46)					1.98 (1.22-2.92)					
	Negative	11	0.88 (0.35-1.29)					2.55 (1.42-3.00)					
**Oligodendrocyte transcription factor 2**					–0.74^a^		.46				0.15^a^		.89
	Positive	153	0.88 (0.58-1.46)					2.03 (1.22-2.92)					
	Negative	7	0.83 (0.55-0.94)					1.89 (1.76-2.54)					
**P53 protein**					1.08^a^		.28				0.58^a^		.57
	Positive	142	0.87 (0.58-1.43)					1.98 (1.22-2.89)					
	Negative	18	1.13 (0.55-2.36)					1.97 (1.33-3.18)					
**Alpha-thalassemia/mental retardation syndrome X-linked**					1.71^a^		.09				1.48^a^		.14
	Positive	133	0.85 (0.54-1.43)					1.94 (1.22-2.76)					
	Negative	27	1.13 (0.70-2.35)					2.73 (1.40-3.55)					
**Cluster of differentiation 34**					–0.39^a^		.70				–0.47^a^		.64
	Positive	51	0.85 (0.60-1.57)					2.07 (1.34-3.03)					
	Negative	109	0.89 (0.51-1.43)					1.95 (1.22-2.89)					
**Epithelial membrane antigen**					–1.27^a^		.20				–0.51^a^		.61
	Positive	15	1.02 (0.85-1.59)					1.81 (1.45-3.29)					
	Negative	145	0.86 (0.54-1.46)					2.03 (1.22-2.92)					
**Synuclein**					2.86^a^		.004				2.63^a^		.009
	Positive	35	0.67 (0.35-0.92)					1.44 (1.01-2.39)					
	Negative	125	0.91 (0.64-1.54)					2.15 (1.35-3.14)					
**Neuronal nuclei**					–1.11^a^		.27				–0.35^a^		.73
	Positive	3	1.72 (1.27-1.83)					1.96 (1.95-2.35)					
	Negative	157	0.88 (0.55-1.46)					1.99 (1.22-2.94)					
**O6-methylguanine-DNA methyltransferase promoter methylation**					–2.86^a^		.004				–2.09^a^		.04
	Positive	8	1.96 (1.25-2.44)					2.87 (2.16-3.63)					
	Negative	152	0.86 (0.54-1.43)					1.94 (1.22-2.90)					
**Isocitrate dehydrogenase 1 R132**					–4.57^a^		<.001				–4.36^a^		<.001
	Positive	16	1.88 (1.39-3.10)					3.34 (2.84-3.82)					
	Negative	144	0.85 (0.51-1.23)					1.85 (1.21-2.72)					
**1p loss of heterozygosity**					–1.98^a^		.049				–3.00^a^		.003
	positive	6	2.08 (1.19-2.52)					3.69 (3.31-4.27)					
	negative	154	0.87 (0.56-1.43)					1.94 (1.22-2.89)					
**19q loss of heterozygosity**					–3.76^a^		<.001				–3.69^a^		<.001
	positive	11	1.79 (1.32-3.73)					3.68 (2.63-4.19)					
	negative	149	0.85 (0.53-1.42)					1.89 (1.21-2.84)					

^a^z values determined using the Wilcoxon rank sum test with two-group comparisons.

^b^H values determined using the Kruskal-Wallis test with multiple-group comparisons.

### Construction and Validation of an OS-Based Prediction Model

#### Feature Variable Selection

Using OS as the outcome variable, a total of 3 machine learning algorithms—LASSO regression, XGBoost, and RF—were used to screen feature variables.

**Figure 3:**
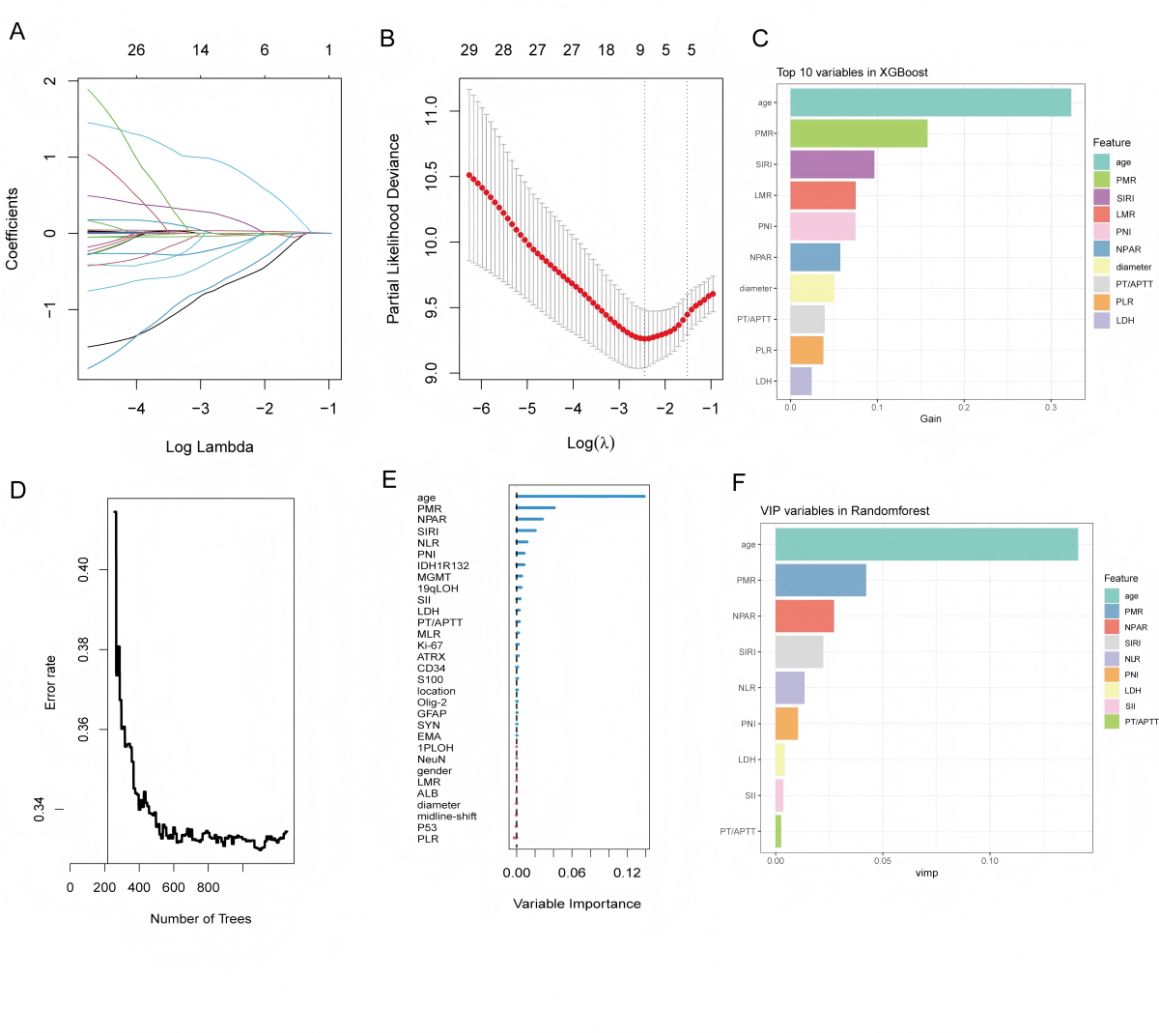
Feature variable selection based on 3 machine learning methods: (A) least absolute shrinkage and selection operator (LASSO) regression coefficient path plot: variable coefficients as a function of λ; (B) LASSO regression λ value tuning: 10-fold cross-validation to select the minimum λ value (λ_min); (C) extreme gradient boosting (XGBoost) feature importance: top 10 variables; (D) random forest (RF) error rate change: error rate as a function of the number of decision trees; (E) feature importance: assessed by Gini index; and (F) RF selected variables: top 9 variables. 19qLOH: 19q loss of heterozygosity; 1pLOH: 1p loss of heterozygosity; ALB: albumin; ATRX: alpha-thalassemia/mental retardation syndrome X-linked; CD34: cluster of differentiation 34; EMA: epithelial membrane antigen; GFAP: glial fibrillary acidic protein; IDH1R132: isocitrate dehydrogenase 1 R132; Ki-67: proliferation cell nuclear antigen-67; LDH: lactate dehydrogenase; LMR: lymphocyte-to-monocyte ratio; MGMT: O6-methylguanine-DNA methyltransferase; MLR: monocyte-to-lymphocyte ratio; NeuN: neuronal nuclei; NLR: neutrophil-to-lymphocyte ratio; NPAR: neutrophil percentage-to-albumin ratio; Olig-2: oligodendrocyte transcription factor 2; P53: P53 protein; PLR: platelet-to-lymphocyte ratio; PMR: platelet-to-mean platelet volume ratio; PNI: prognostic nutritional index; PT/APTT: prothrombin time/activated partial thromboplastin time; S100: S100 calcium-binding protein; SII: immune-inflammation index; SIRI: systemic inflammation response index; SYN: synuclein.

LASSO regression: based on the minimum λ value (λ_min), a total of 9 variables with nonzero coefficients were selected, including age, NPAR, platelet-to-mean platelet volume ratio (PMR), CD34 expression, SYN expression, EMA expression, IDH1 R132, 19qLOH, and MGMT promoter methylation (Figures 3A and B).

XGBoost model: according to feature importance scores, a total of 10 variables were selected, including age, PMR, SIRI, LMR, PNI, NPAR, maximum tumor diameter, PT/APTT, PLR, and LDH (Figure 3C).

RF: based on the mean decrease in Gini index, a total of 9 variables were selected, including age, PMR, NPAR, SIRI, NLR, PNI, LDH, SII, and PT/APTT (Figures 3D-F).

#### Survival Analysis

**Figure 4:**
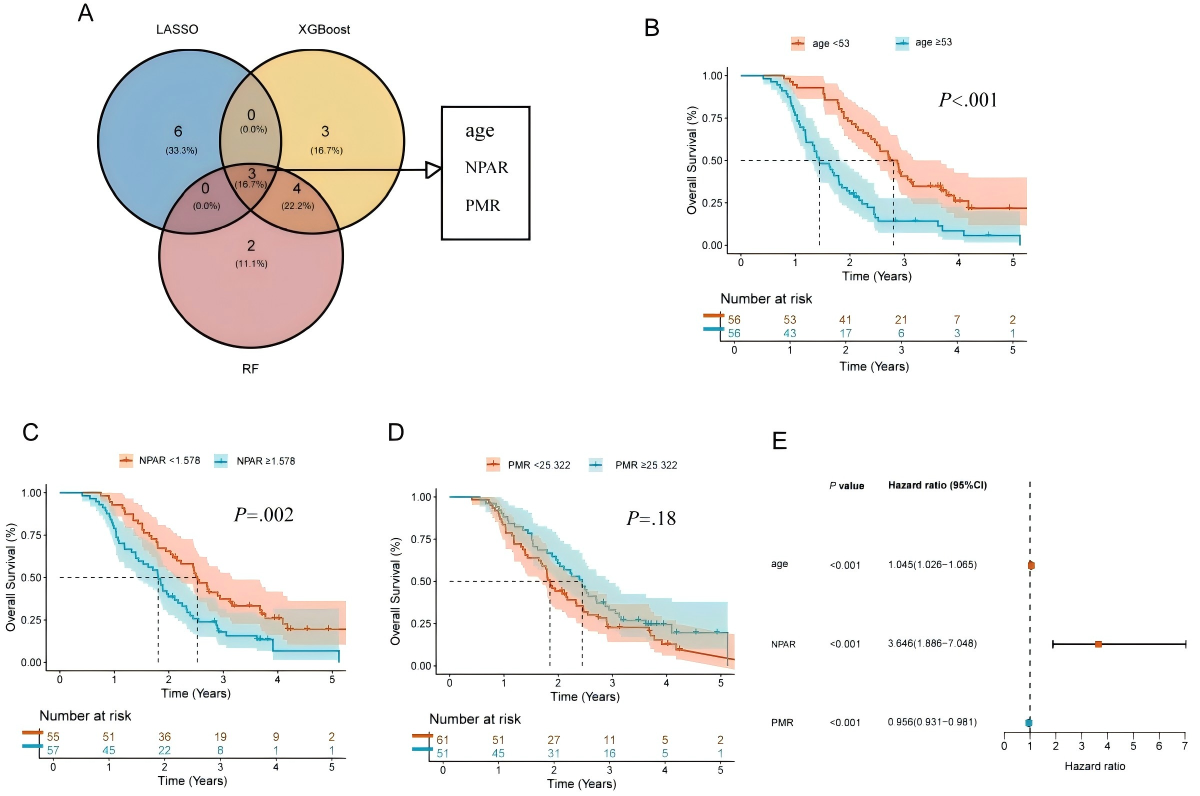
Identification of independent prognostic factors for overall survival (OS): (A) intersection analysis of feature variables from 3 models, (B) impact of age group on patient OS, (C) impact of neutrophil percentage-to-albumin ratio (NPAR) level on patient OS, (D) impact of platelet-to-mean platelet volume ratio (PMR) level on patient OS, and (E) forest plot of multivariate Cox regression analysis for OS. LASSO: least absolute shrinkage and selection operator.

Based on the combined screening results from LASSO, XGBoost, and RF, Venn diagram intersection analysis identified age, NPAR, and PMR as key factors influencing OS (Figure 4A).

The prognostic impact of each individual variable was assessed using KM survival analysis (Figures 4B-D). Log-rank test results indicated that patients aged <53 years had significantly longer OS compared to those aged ≥53 years (*P*<.001), patients with NPAR <1.578 had significantly better OS than those with NPAR ≥1.578 (*P*<.001), while no significant difference in OS was observed between patients with PMR <25.322 and those with PMR ≥25.322 (*P*=.18).

Multivariate Cox proportional hazards regression analysis identified age (HR 1.05, 95% CI 1.03-1.07; *P*<.001), NPAR (HR 3.65, 95% CI 1.89-7.05; *P*<.001), and PMR (HR 0.96, 95% CI 0.93-0.98; *P*<.001) as independent prognostic factors for OS.

#### Construction and Validation of the OS Nomogram

**Figure 5:**
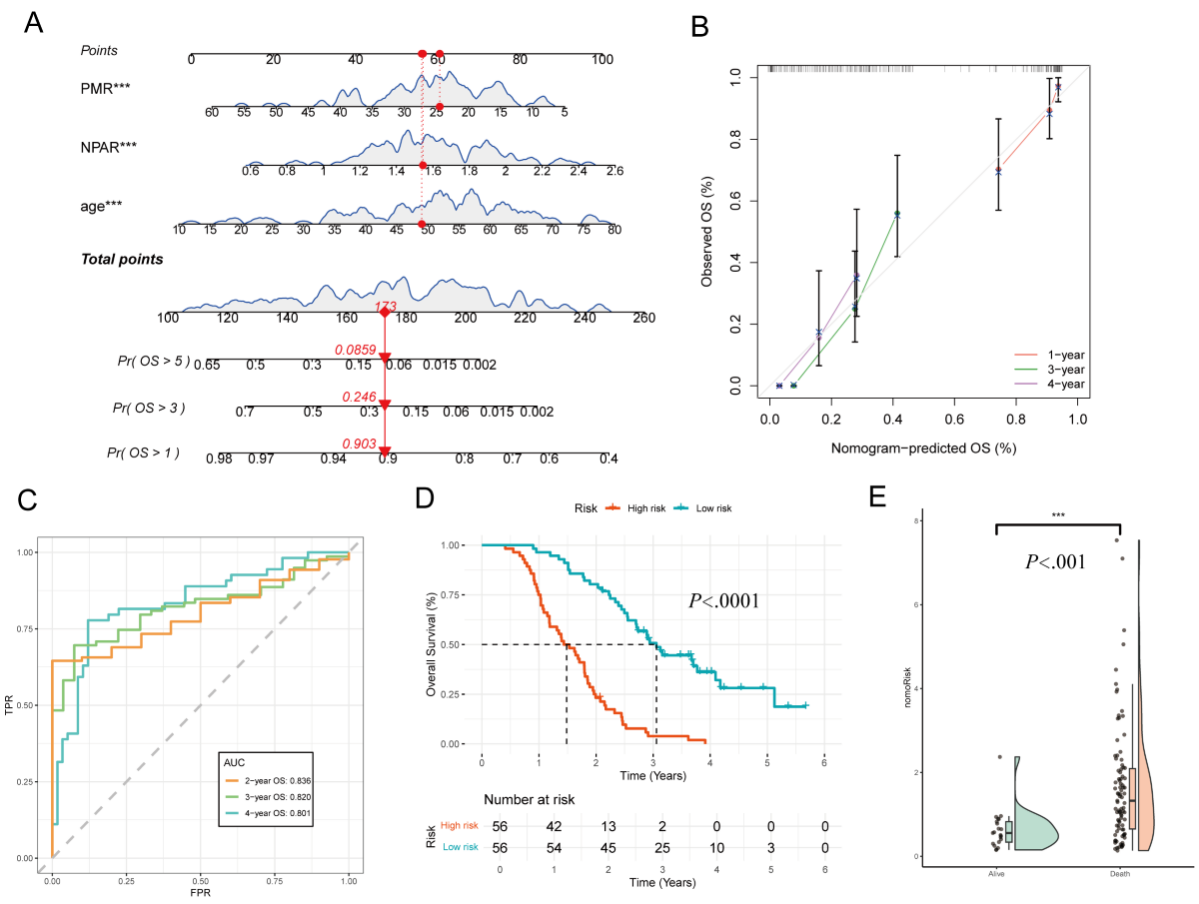
Predictive performance of the overall survival (OS) nomogram: (A) prognostic nomogram integrating age, neutrophil percentage-to-albumin ratio (NPAR), and platelet-to-mean platelet volume ratio (PMR) to predict 1-, 3-, and 5-year OS probabilities through score conversion. For individual prediction, assign points for each variable, sum them to obtain a total score, and project this score downward to the bottom probability axes; (B) the calibration curve demonstrates the agreement between predicted and observed 1-, 3-, and 4-year OS probabilities in the training group; (C) time-dependent receiver operating characteristic (ROC) curves show area under the curve (AUC) values of 0.836, 0.820, and 0.801 for 2-, 3-, and 4-year OS, respectively; (D) survival analysis based on the median nomogram score reveals significant differences in OS between the high-risk and low-risk groups (P<.001); and (E) raincloud plot comparing the distribution of nomogram scores between the survival and death groups.

Based on the multivariate Cox regression analysis, age, NPAR, and PMR were identified as independent prognostic factors for OS. A nomogram was constructed using these variables to predict 1-, 3-, and 5-year OS probabilities. Each variable was assigned a corresponding risk score, and the total score was used to estimate individual survival probability (Figure 5A). The calibration curve showed good agreement between the predicted and observed 1-, 3-, and 5-year OS rates (Figure 5B). Time-dependent ROC analysis demonstrated that the AUCs for 2-, 3-, and 4-year OS in the training cohort were 0.836, 0.820, and 0.801, respectively, indicating strong predictive performance across multiple timepoints (Figure 5C).

Patients were stratified into high- and low-risk groups based on the median nomogram score. KM survival analysis revealed that the high-risk group had significantly poorer OS compared with the low-risk group (*P*<.001), supporting the nomogram’s utility in risk stratification (Figure 5D).

To further evaluate the discriminative ability of the nomogram score, a raincloud plot was used to visualize the score distribution between the survival and death groups. This plot, which integrates a density plot, box plot, and scatter plot, clearly illustrated the differences in score distribution, providing additional evidence of the nomogram’s effectiveness in prognostic assessment (Figure 5E).

**Figure 6:**
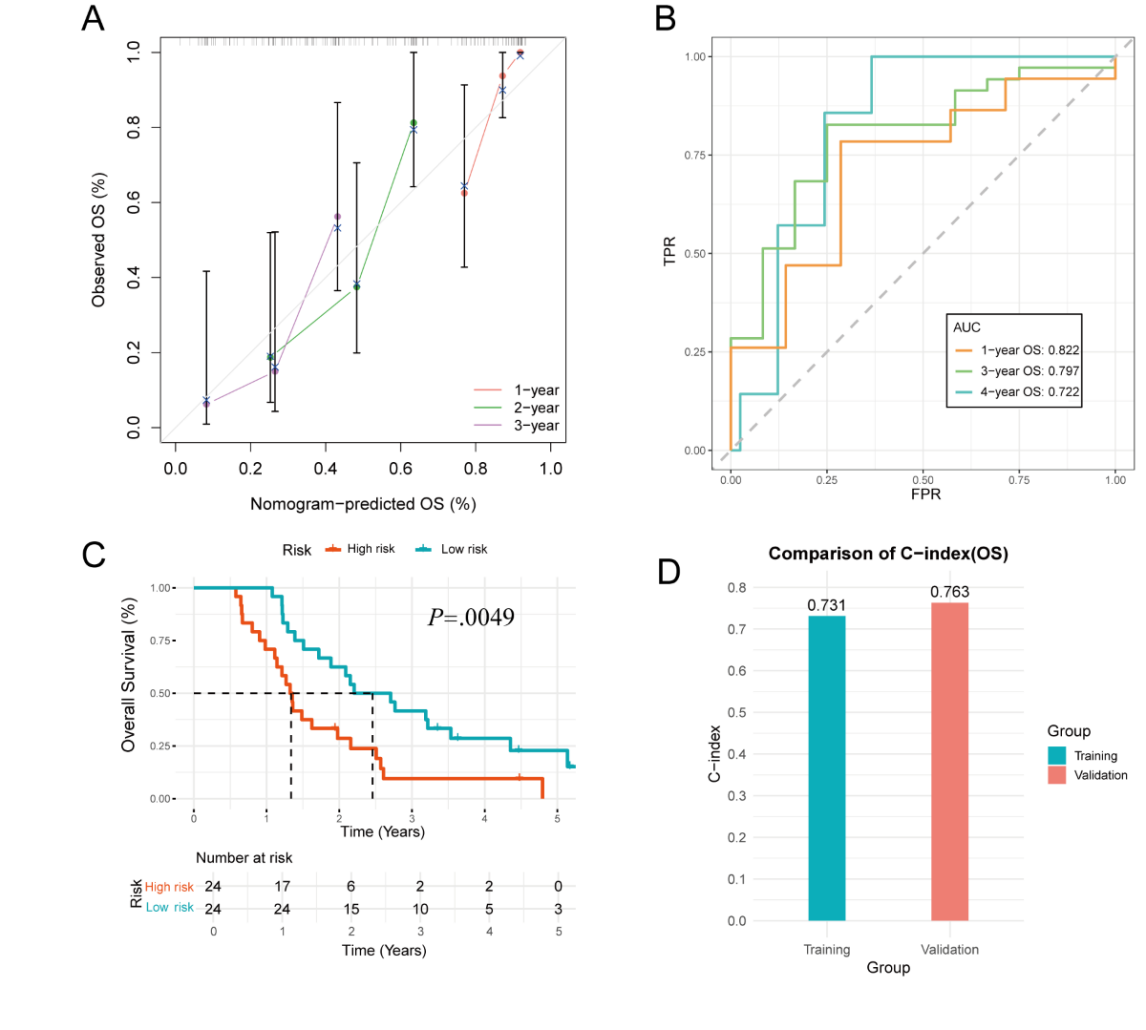
Validation of the overall survival (OS) nomogram: (A) the calibration curve demonstrates the agreement between predicted and observed 1-, 2-, and 3-year OS probabilities in the validation group; (B) time-dependent receiver operating characteristic (ROC) curves show area under the curve (AUC) values of 0.822, 0.797, and 0.722 for 1-, 3-, and 4-year OS, respectively; (C) survival analysis based on the median nomogram score reveals significant differences in OS between the high-risk and low-risk groups; and (D) comparison of the concordance index (C-index) between the training and validation groups.

The calibration curve in the validation group indicated good predictive accuracy of the model for 1-, 2-, and 3-year survival in patients with ADG (Figure 6A). Time-dependent ROC analysis showed that the AUCs for 1-, 3-, and 4-year OS were 0.822, 0.797, and 0.722, respectively (Figure 6B). Risk stratification based on the nomogram score revealed a significant difference in score distribution between survivors and nonsurvivors. Patients in the high-risk group had significantly poorer OS compared with those in the low-risk group (*P*<.01), supporting the prognostic discriminatory power of the nomogram (Figure 6C). The C-index remained high in both the training and validation sets, suggesting robust and consistent predictive performance (Figure 6D).

### Construction and Validation of the PFS Prediction Model for ADG

#### Feature Variable Selection

Using PFS as the outcome variable, 3 machine learning algorithms—LASSO regression, XGBoost, and RF—were used to screen feature variables.

**Figure 7:**
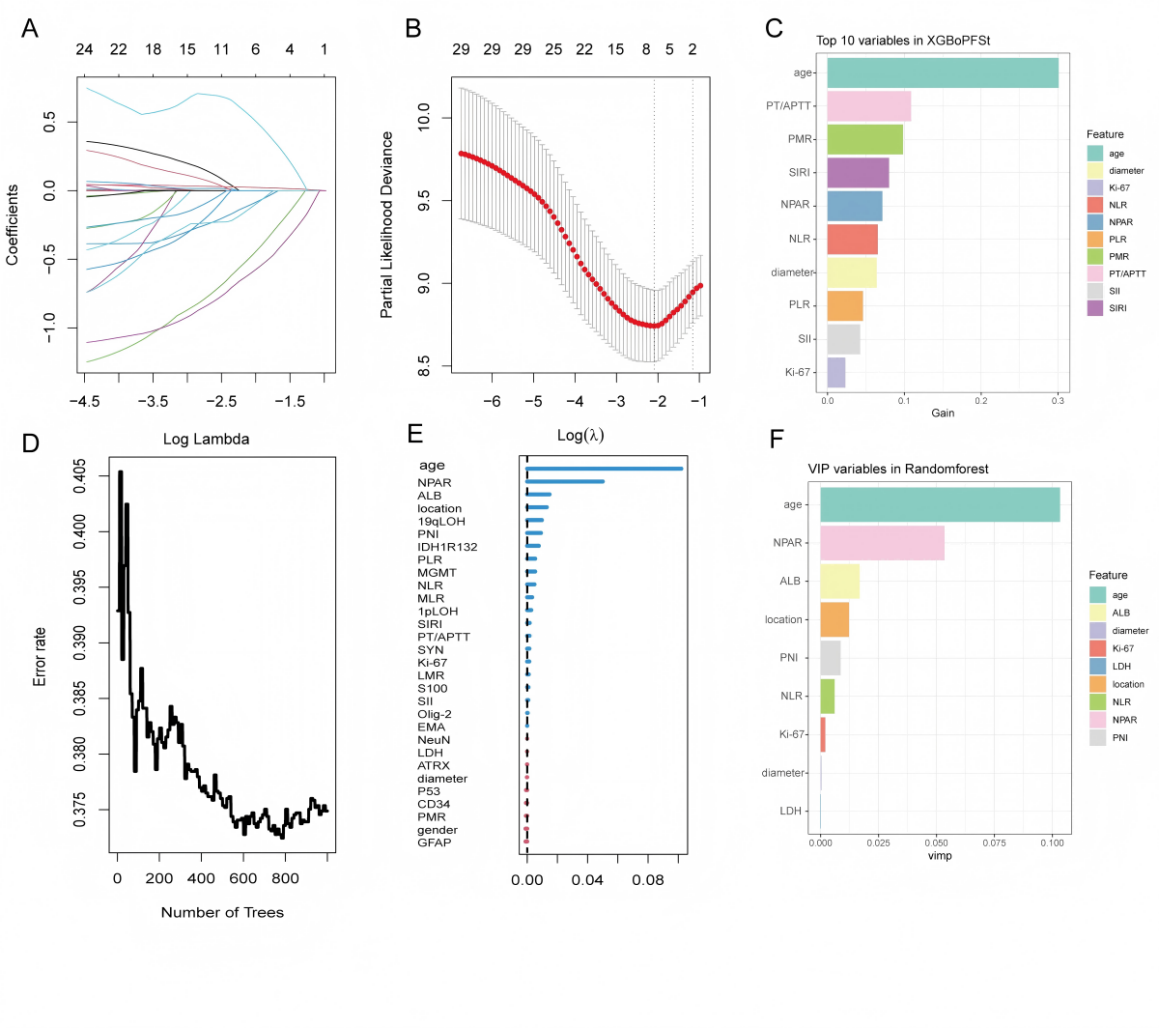
Feature variable selection based on 3 machine learning methods: (A) least absolute shrinkage and selection operator (LASSO) regression coefficient path plot: variable coefficients as a function of λ, (B) LASSO regression parameter λ tuning based on the minimum criterion, (C) top 10 variables ranked by importance in the extreme gradient boosting (XGBoost) model, (D) change in error rate as a function of the number of decision trees during random forest (RF) model training, (E) RF feature importance: assessed by Gini index, and (F) RF selected variables: top 9 variables. 19qLOH: 19q loss of heterozygosity; 1pLOH: 1p loss of heterozygosity; ALB: albumin; ATRX: alpha-thalassemia/mental retardation syndrome X-linked; CD34: cluster of differentiation 34; EMA: epithelial membrane antigen; GFAP: glial fibrillary acidic protein; IDH1R132: isocitrate dehydrogenase 1 R132; Ki-67: proliferation cell nuclear antigen-67; LDH: lactate dehydrogenase; LMR: lymphocyte-to-monocyte ratio; MGMT: O6-methylguanine-DNA methyltransferase; MLR: monocyte-to-lymphocyte ratio; NeuN: neuronal nuclei; NLR: neutrophil-to-lymphocyte ratio; NPAR: neutrophil percentage-to-albumin ratio; Olig-2: oligodendrocyte transcription factor 2; P53: P53 protein; PLR: platelet-to-lymphocyte ratio; PMR: platelet-to-mean platelet volume ratio; PNI: prognostic nutritional index; PT/APTT: prothrombin time/activated partial thromboplastin time; S100: S100 calcium-binding protein; SII: immune-inflammation index; SIRI: systemic inflammation response index; SYN: synuclein.

LASSO regression: based on the minimum λ value (λ_min), a total of 6 nonzero coefficient variables were selected, including age, NPAR, MLR, MGMT methylation, 1pLOH, and 19qLOH (Figure 7A and B).

XGBoost model: based on feature importance scores, a total of 10 variables were selected, including age, PT/APTT, PMR, SIRI, NPAR, NLR, maximum tumor diameter, PLR, SII, and Ki-67 expression (Figure 7C).

RF: based on the mean decrease in Gini index, a total of 9 variables were selected, including age, NPAR, ALB, tumor location, PNI, NLR, Ki-67, maximum tumor diameter, and LDH (Figures 7D and E).

#### Survival Analysis

**Figure 8:**
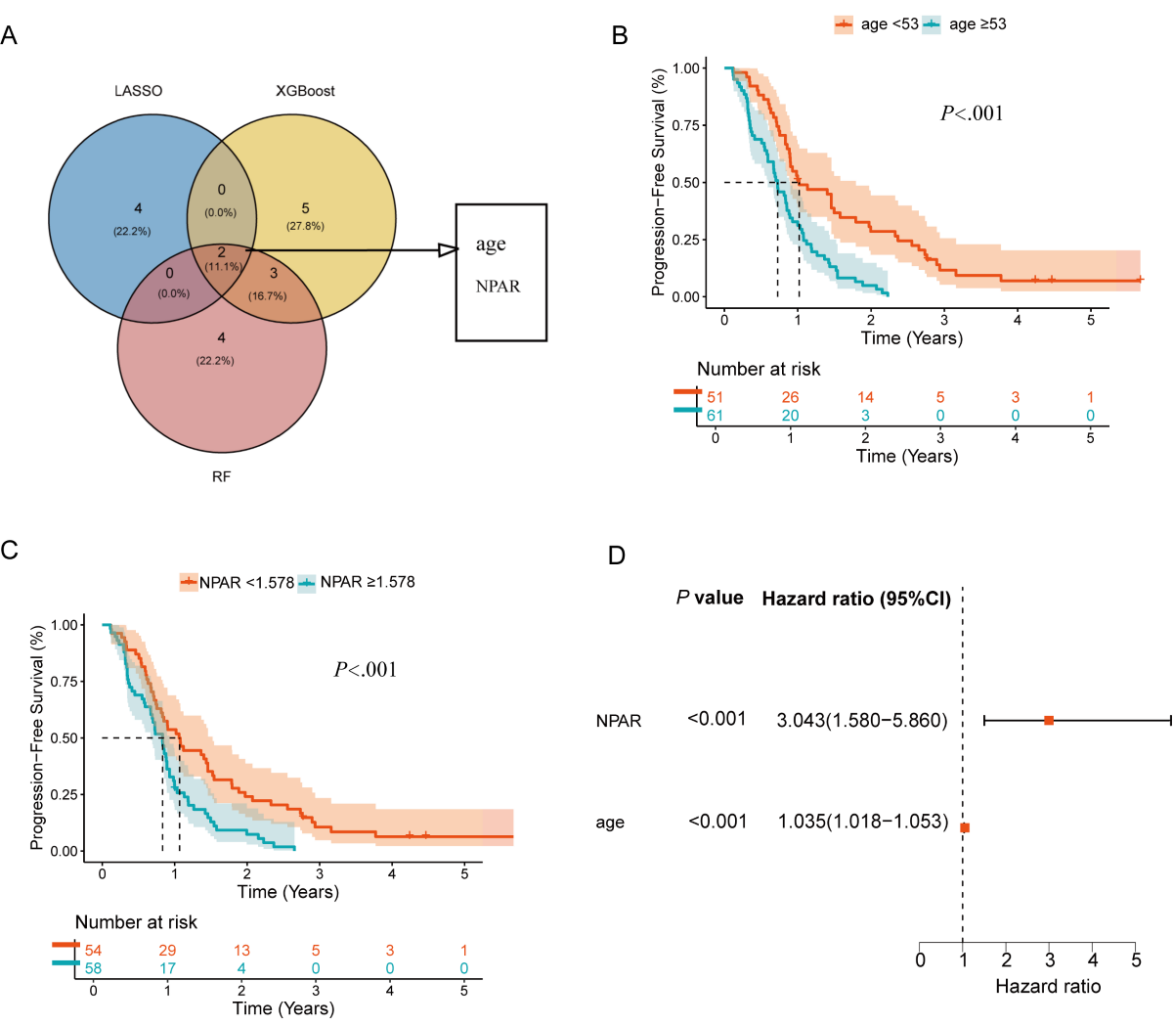
Identification of independent prognostic factors for progression-free survival (PFS): (A) intersection analysis of feature variables from 3 models, (B) impact of age group on patient PFS, (C) impact of neutrophil percentage-to-albumin ratio (NPAR) level on patient PFS, and (D) forest plot of multivariate Cox regression analysis for PFS. LASSO: least absolute shrinkage and selection operator.

Based on the important variables screened by LASSO, XGBoost, and RF, Venn diagram intersection analysis identified age and NPAR as key factors influencing PFS (Figure 8A). The impact of each single factor on prognosis was assessed using KM survival analysis (Figures 8B and C). Log-rank test results showed that patients in the age <53 years group had significantly longer PFS than those in the age ≥53 years group (*P*<.001); patients in the NPAR <1.578 group had significantly longer PFS than those in the NPAR ≥1.578 group (*P*<.001). Multivariate Cox proportional hazards regression analysis revealed that age (HR 1.04, 95% CI 1.02-1.05; *P*<.001) and NPAR (HR 3.04, 95% CI 1.58-5.86; *P*<.001) were independent prognostic factors for PFS (Figure 8D).

#### Construction and Validation of the PFS Nomogram

**Figure 9:**
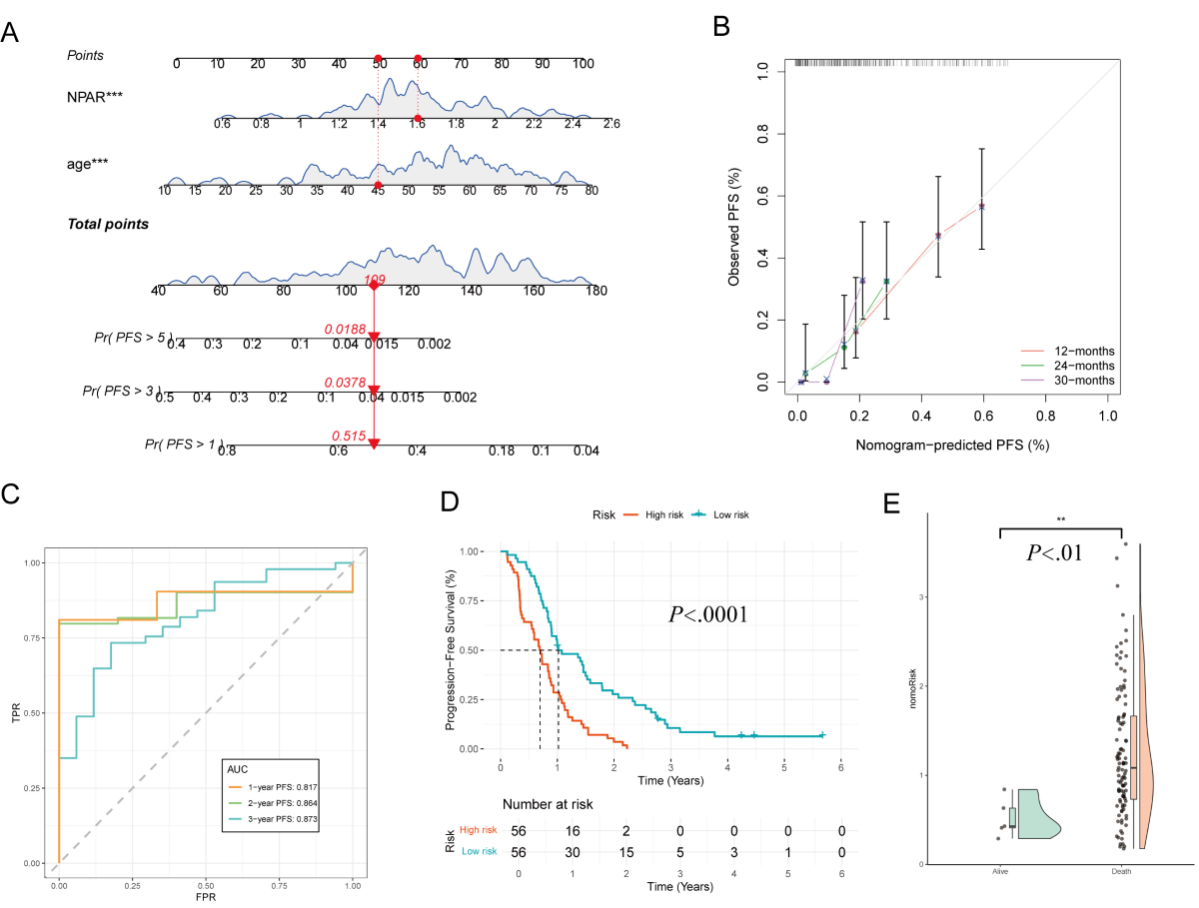
Predictive performance of the progression-free survival (PFS) nomogram: (A) prognostic nomogram integrating age and NPAR to predict 1-, 3-, and 5-year PFS probabilities through score conversion. For individual prediction, assign points for each variable, sum them to obtain a total score, and project this score downward to the bottom probability axes. (B) The calibration curve demonstrates the agreement between predicted and observed 12-, 24-, and 30-month PFS probabilities in the training group. (C) Time-dependent receiver operating characteristic (ROC) curves show area under the curve (AUC) values of 0.817, 0.864, and 0.873 for 1-, 2-, and 3-year PFS, respectively. (D) Survival analysis based on the median nomogram score reveals significant differences in PFS between the high-risk and low-risk groups (P<.001). (E) Raincloud plot comparing the distribution of nomogram scores between the survival (alive) and death (death) groups.

Based on multivariate Cox regression analysis, age and NPAR were identified as independent prognostic factors for PFS. A nomogram was constructed using these variables to predict 1-, 3-, and 5-year PFS rates. Each predictive variable was assigned a corresponding risk score, and the total score estimated the patient’s probability of disease progression (Figure 9A).

The calibration curve demonstrated good agreement between the model-predicted and observed 12-, 24-, and 30-month PFS rates (Figure 9B). Time-dependent ROC curve analysis showed that the AUC for 1-, 2-, and 3-year PFS in the training group was 0.817, 0.864, and 0.873, respectively, indicating high predictive accuracy of the model at different timepoints (Figure 9C). Patients were divided into high-risk and low-risk groups based on the median nomogram score. KM survival curves revealed that the high-risk group had significantly lower PFS than the low-risk group (*P*<.001), demonstrating that the nomogram score effectively stratified patients’ prognostic risk (Figure 9D).

The raincloud plot provides a visual representation of the score distribution differences between the 2 groups and further supporting the effectiveness of the nomogram score in prognostic assessment (Figure 9E).

**Figure 10:**
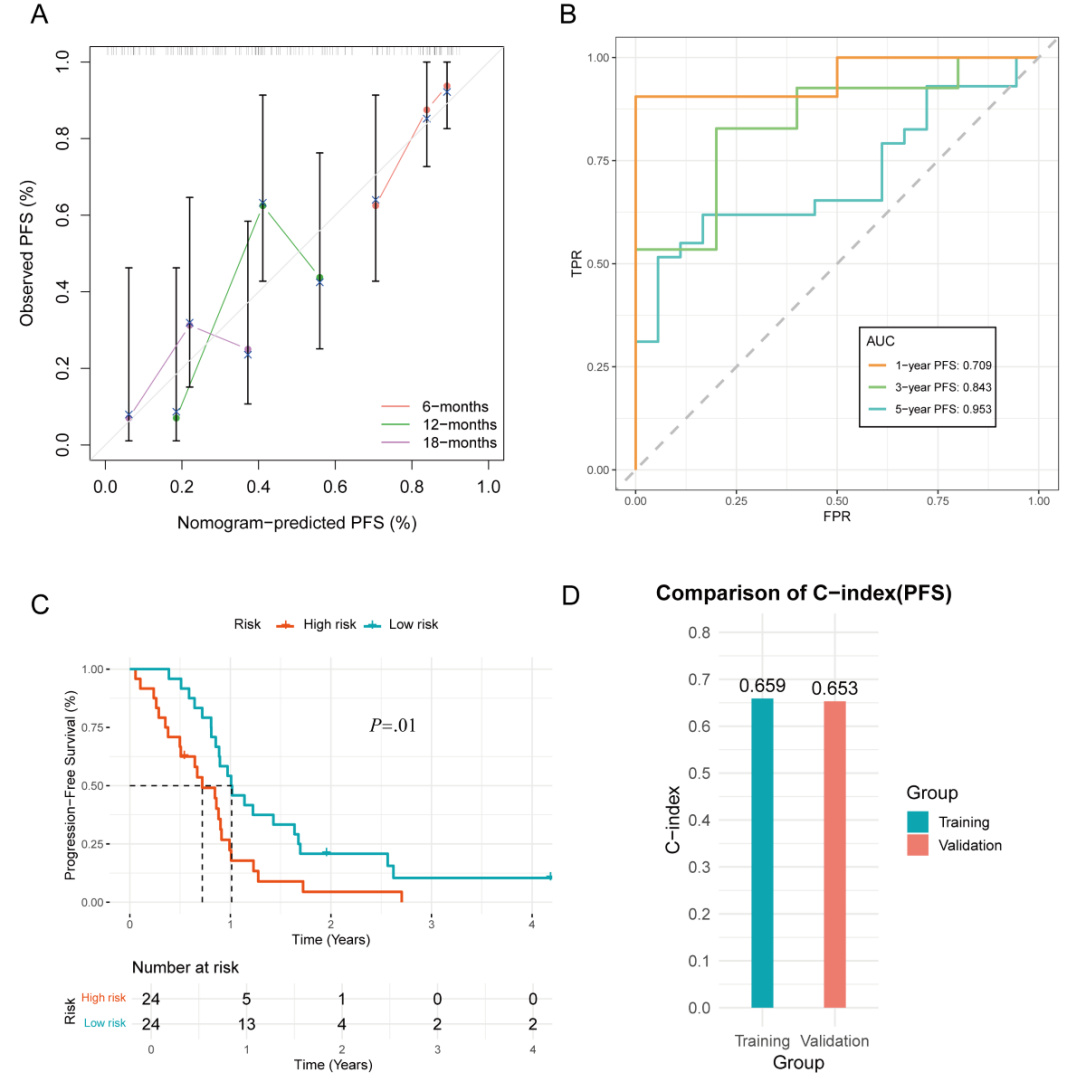
Validation of the progression-free survival (PFS) nomogram: (A) the calibration curve demonstrates the agreement between predicted and observed 6-, 12-, and 18-month PFS probabilities in the validation group; (B) time-dependent receiver operating characteristic (ROC) curves show area under the curve (AUC) values of 0.709, 0.843, and 0.953 for 1-, 3-, and 5-year PFS, respectively; (C) survival analysis based on the median nomogram score reveals significant differences in PFS between the high-risk and low-risk groups; and (D) comparison of the concordance index (C-index) between the training and validation groups.

Due to the high risk of patient recurrence, the calibration curve for the validation group demonstrated good predictive performance for 6-, 12-, and 18-month disease progression risk in patients with ADG (Figure 10A). Time-dependent ROC curve analysis showed that the AUC for 1-, 3-, and 5-year PFS in the validation group was 0.709, 0.843, and 0.953, respectively (Figure 10B). Based on the median nomogram score, patients in the validation group were divided into high-risk and low-risk groups. KM survival curves revealed significant differences in OS between the 2 groups (*P*<.05), further validating the prognostic discriminative ability of the nomogram score (Figure 10C).

The C-index of the model was excellent in both the training and validation groups, indicating stable predictive performance across different datasets (Figure 10D).

## Discussion

### Principal Results

ADG, characterized by high malignancy and strong invasiveness, accounts for 45% of all gliomas [20,21]. The tumor heterogeneity and infiltrative growth patterns of ADG often lead to progressive neurological impairment, posing significant challenges for clinical management [9,22]. This study demonstrated that standardized comprehensive treatment (surgery combined with concurrent chemoradiotherapy and temozolomide) extended the median overall survival (mOS) to 23.0 (IQR 15.0-38.3) months, which is superior to the approximately 15 months reported in previous studies [2,23,24]. However, 95% (152/160) of patients experienced postoperative recurrence, with a median progression-free survival (mPFS) of only 10.8 months and a 5-year PFS rate of merely 1.9% (3/160). Moreover, the long-term prognosis remains poor, with a 5-year OS rate of just 12.8% (20/160) [25]. Therefore, it is of great significance to further explore the key prognostic factors affecting patients with ADG, establish novel prognostic models, and develop effective preventive strategies.

The key methodological advance of our work lies in the robust feature selection achieved by integrating 3 distinct machine learning algorithms (LASSO, XGBoost, and RF) [26]. This integrative approach circumvents the inherent pitfalls of relying on a single method, be it traditional Cox regression or an individual machine learning model [13,14]. Consequently, it ensures that the identified prognostic factors (age, NPAR, and PMR) are consistently salient across different modeling paradigms, thereby significantly strengthening the credibility of our results.

Compared to existing prognostic models for ADG, our model demonstrates superior predictive performance. While Kim et al [27] reported a C-index of 0.70 using a single machine learning approach, the OS model in this study achieved C-indices of 0.731 and 0.763 in the training and validation cohorts, respectively. Furthermore, the model maintained high time-dependent AUC values at multiple timepoints (AUCs of 0.836, 0.820, and 0.801 for 2-, 3-, and 4-year OS, respectively) along with favorable calibration. Importantly, our model innovatively incorporates readily accessible hematological biomarkers (NPAR and PMR). This provides a more cost-effective and clinically feasible prognostic assessment tool compared to traditional models that rely solely on clinical and molecular parameters [7,8].

Analysis of 32 variables showed that longer survival was observed in patients with canonical molecular markers such as IDH1 mutation and MGMT promoter methylation, implicating them as potential prognostic factors. IDH1 mutations play a critical role in suppressing tumor metabolism and proliferation [28,29]. Patients with IDH1-mutant astrocytoma included had an mOS of 40.0 (IQR 33.6–NA, NA is censored data—some patients remained alive at follow-up cutoff, so the upper quartile was not observed) months, markedly better than the 22.2 (IQR 14.4–34.7) months observed in IDH1 wild-type patients. Similarly, MGMT methylation enhances sensitivity of tumor cells to alkylating agents such as temozolomide by inhibiting MGMT transcription and reducing DNA repair capacity, thereby significantly prolonging both PFS and OS [29-31]. In this study, a 30-year-old patient with IDH-mutant astrocytoma and MGMT methylation achieved a survival period exceeding 64 months, demonstrating significant survival benefit [32].

Furthermore, by integrating multiple machine learning methods, the study systematically identified age, the inflammation-nutrition marker NPAR, and the platelet quantity and function marker PMR as independent prognostic factors for patients with ADG [16]. These variables demonstrated significant predictive power in forecasting the prognosis of patients with ADG [33,34].

Age was confirmed as a key prognostic factor in our model. Patients aged ≥53 years had a higher risk of disease progression and significantly shorter survival time compared with those aged <53 years. This finding aligns with previous research trends regarding the prognosis of older adults with ADG, which may be related to reduced treatment tolerance and alterations in tumor biological characteristics in older individuals. It suggests that clinicians should pay attention to age stratification and develop personalized treatment plans accordingly [28].

This study is the first to identify NPAR as an independent prognostic factor for ADG. Patients with high NPAR levels had significantly shorter mOS and mPFS, along with a markedly increased risk of disease recurrence and worse prognosis. As a novel indicator that comprehensively reflects systemic inflammation and nutritional status, the NPAR has increasingly been recognized for its prognostic value in various cancers such as pancreatic and urological cancers in recent years. The NPAR integrates both systemic inflammation (reflected by neutrophil percentage) and nutritional status (reflected by serum ALB), 2 key pathophysiological processes known to influence cancer prognosis. Neutrophils can impair immune surveillance by suppressing natural killer cell function and promote tumor cell proliferation and metastasis [12,35]. Serum ALB levels not only reflect the body’s protein metabolic status but are also closely associated with immune dysfunction [36]. The prognostic value of NPAR may stem from its correlation with systemic inflammatory responses mediated by proinflammatory cytokines in the TME. This pathophysiological cascade can accelerate the progression of cachexia and facilitate the evolution of malignant tumor phenotypes by modulating the TME [37,38].

The prognostic value of NPAR stems from its integration of 2 pivotal pathophysiological pathways: protumor inflammation and host nutritional status. The neutrophil component reflects systemic inflammation. Within the glioma TME, neutrophils are polarized into tumor-associated neutrophils (TANs), which facilitate tumor progression by secreting matrix metalloproteinases to enhance invasion, releasing proangiogenic factors like vascular endothelial growth factor, and contributing to T-cell dysfunction [39]. Emerging evidence specifically links this protumorigenic neutrophil activity to glioma progression [40]. Conversely, hypoalbuminemia, indicative of poor nutritional status and diminished physiological reserve, is a well-established marker of impaired immune competence and poorer outcomes in patients with cancer [41]. Thus, NPAR comprehensively captures this detrimental interplay between a proinflammatory TME and a compromised host state.

Furthermore, this study identified a higher PMR as an independent prognostic factor for prolonged overall survival in patients with ADG. This finding is consistent with a study on angiosarcoma, which also reported that a high mean platelet volume–to–platelet count ratio served as an independent risk factor for patient mortality [42]. This consistent pattern across malignancies highlights the biological significance of composite indices based on platelet count and volume.

While studies have established that platelets can facilitate glioma progression by releasing factors like platelet-derived growth factor to stimulate proliferation [43] and by modulating the immune microenvironment to promote an M2 phenotype and immune escape [44], our discovery of PMR as a protective factor suggests a more nuanced role. We hypothesize that an elevated PMR reflects a distinct platelet phenotype characterized by a lower mean platelet volume which is generally associated with diminished platelet reactivity. Consequently, a high PMR may indicate a reduced capacity of platelets to engage in these documented protumorigenic processes, thereby explaining its association with improved survival in ADG.

The prognostic nomogram developed from the aforementioned factors demonstrates significant translational potential. This tool can be integrated into electronic medical record systems to enable automated risk stratification at the time of diagnosis. Consequently, high-risk patients could be considered for more aggressive treatment strategies—such as intensified chemoradiation following maximal safe resection—or more intensive follow-up (eg, neuroimaging every 2-3 months), and even enrollment in novel clinical trials. Conversely, low-risk patients may be suitable candidates for treatment de-escalation, such as shortened adjuvant chemotherapy cycles or reduced radiation doses, aiming to preserve oncological control while enhancing quality of life. This nomogram-based decision-making model represents a constructive step toward precision neuro-oncology.

### Limitations

This study is subject to several limitations, including its single-center retrospective design, which may limit generalizability, and a variable-to-sample size ratio that raises concerns about overfitting despite internal validation. These limitations underscore the necessity for external validation in independent multicenter cohorts as an essential next step. Moreover, the molecular mechanisms linking NPAR and PMR to glioma prognosis are not fully understood. To address these points and advance this research, future work must focus on multicenter prospective validation, augmenting the model’s power through the integration of radiomics and genomics, and leveraging single-cell and multi-omics technologies to decipher the functional roles of these circulating biomarkers in glioma pathogenesis.

### Conclusion

In conclusion, this study significantly improved the robustness of prognostic biomarkers through a multimodel cross-validation strategy. Among the numerous factors, age, the preoperative inflammation-nutrition composite indicator NPAR, and platelet quantity and function marker PMR were key prognostic factors. Patients with ADG with younger age and lower NPAR values had a lower risk of disease progression and better prognosis; meanwhile, patients with higher PMR exhibited longer survival. These biomarkers are not only easily accessible and cost-effective but also highly beneficial, offering a new perspective for personalized treatment of ADG.

## Abbreviations

19qLOH: 19q loss of heterozygosity
1pLOH: 1p loss of heterozygosity
ADG: adult-type diffuse glioma
AISI: aggregate index of systemic inflammation
ALB: albumin
APTT: activated partial thromboplastin time
ATRX: alpha-thalassemia/mental retardation syndrome X-linked
AUC: area under the curve
BRAF: B-Raf proto-oncogene, serine/threonine kinase
CD34: cluster of differentiation 34
C-index: concordance index
EGFR: epidermal growth factor receptor
EMA: epithelial membrane antigen
GFAP: glial fibrillary acidic protein
HR: hazard ratio
IDH1: isocitrate dehydrogenase 1
Ki-67: proliferation cell nuclear antigen-67
KM: Kaplan-Meier
LASSO: least absolute shrinkage and selection operator
LDH: lactate dehydrogenase
LMR: lymphocyte-to-monocyte ratio
Lym: lymphocyte
MGMT: O6-methylguanine-DNA methyltransferase
MLR: monocyte-to-lymphocyte ratio
Mono: monocyte
mOS: median overall survival
mPFS: median progression-free survival
NeuN: neuronal nuclei
Neut: neutrophil
NLPR: neutrophil-lymphocyte-platelet ratio
NLR: neutrophil-to-lymphocyte ratio
NPAR: neutrophil percentage–to-albumin ratio
Olig-2: oligodendrocyte transcription factor 2
OS: overall survival
P53: P53 protein
PFS: progression-free survival
PLR: platelet-to-lymphocyte ratio
PLT: platelet
PMR: platelet-to-mean platelet volume ratio
PNI: prognostic nutritional index
PT: prothrombin time
RF: random forest
ROC: receiver operating characteristic
S100: S100 calcium-binding protein
SII: immune-inflammation index
SIRI: systemic inflammation response index
SYN: synuclein
TAN: tumor-associated neutrophil
TERT: telomerase reverse transcriptase
TME: tumor microenvironment
WBC: white blood cell
WHO: World Health Organization
XGBoost: extreme gradient boosting
